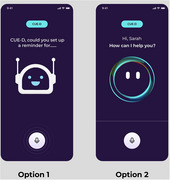# Co‐creating CUE‐D: A Voice Assistant for People Living with Dementia

**DOI:** 10.1002/alz70858_104941

**Published:** 2025-12-26

**Authors:** Erica Dove, Ansh Sharma, Riju Mukherjee, Arlene Astell

**Affiliations:** ^1^ University of Toronto, Toronto, ON, Canada; ^2^ Northumbria University, Newcastle upon Tyne, United Kingdom

## Abstract

**Background:**

Being unable to complete daily activities is a significant factor in people living with dementia losing independence. Timely and appropriate cueing integrated into the physical space can nudge people with dementia back on track to complete activities. We are currently co‐creating a voice assistant to deliver a bespoke cueing strategy for individuals living with dementia using large language models. Currently, we are co‐creating the functionality of the CUE‐D user interface and audio/voice prompts for people living with dementia.

**Method:**

Twenty participants living with dementia plus family carers, are being recruited to test CUE‐D through community‐based user co‐creation sessions. Participants first complete a brief demographic questionnaire capturing sex, education, vision and hearing abilities, plus technology use and experience. Participants are video recorded over the shoulder while interacting with CUE‐D to complete a series of tasks (e.g., asking CUE‐D to set a reminder), to identify any usability and/or functionality issues. After interacting with the app, participants complete the Voice Usability Scale (VUS) and an audio‐recorded interview.

**Result:**

To date, we have iteratively developed and tested CUE‐D with 12 people living with dementia (66.7% male). Participant feedback has iteratively refined our user testing methods and components of the app (e.g., adjusting pauses between user input and the app's voice instructions). VUS scores (with higher scores indicating greater usability) have increased with design updates, and 75% of participants have completed all assigned tasks. One participant shared: *“I would use it for a lot of things around the house.”* We continue to work on recruiting more end users to test the latest adaptations made to CUE‐D

**Conclusion:**

Supporting people with dementia to continue their daily activities can enable individuals to live at home longer and reduce the need for caregiver support. Large language models offer the opportunity to tailor support to the specific needs of individuals living with dementia. This can relieve or delay pressures on families and the formal health and social care systems while respecting the individuality and personhood of people with dementia